# Patterns of 1,748 Unique Human Alloimmune Responses Seen by Simple Machine Learning Algorithms

**DOI:** 10.3389/fimmu.2020.01667

**Published:** 2020-07-28

**Authors:** Angeliki G. Vittoraki, Asimina Fylaktou, Katerina Tarassi, Zafeiris Tsinaris, George Ch. Petasis, Demetris Gerogiannis, Vissal-David Kheav, Maryvonnick Carmagnat, Claudia Lehmann, Ilias Doxiadis, Aliki G. Iniotaki, Ioannis Theodorou

**Affiliations:** ^1^National Tissue Typing Center & Immunology Department, General Hospital of Athens “G.Gennimatas”, Athens, Greece; ^2^National Peripheral Histocompatibility Center – Immunology Department, Hippokration General Hospital, Thessaloniki, Greece; ^3^Immunology-Histocompatibility Department, “Evangelismos” General Hospital, Athens, Greece; ^4^Department of Computer Science and Biomedical Informatics, University of Thessaly, Lamia, Greece; ^5^Department of Computer Science & Engineering, University of Ioannina, Ioannina, Greece; ^6^Laboratoire d'Immunologie, Hôpital St. Louis, Paris, France; ^7^Laboratory for Transplantation Immunology, Institute for Transfusion Medicine, University Hospital Leipzig, Leipzig, Germany; ^8^Nephrology and Transplantation Unit, Medical School of Athens, Laikon Hospital, Athens, Greece; ^9^Centre d'Immunologie et des Maladies Infectieuses UPMC UMRS CR7 - Inserm U1135 - CNRS ERL 8255, Paris, France

**Keywords:** HLA, patterns detection, allorecognition, transplantation, monitoring, PCA, descriptive statistics, machine learning

## Abstract

Allele specific antibody response against the polymorphic system of HLA is the allogeneic response marker determining the immunological risk for graft acceptance before and after organ transplantation and therefore routinely studied during the patient's workup. Experimentally, bead bound antigen- antibody reactions are detected using a special multicolor flow cytometer (Luminex). Routinely for each sample, antibody responses against 96 different HLA antigen groups are measured simultaneously and a 96-dimensional immune response vector is created. Under a common experimental protocol, using unsupervised clustering algorithms, we analyzed these immune intensity vectors of anti HLA class II responses from a dataset of 1,748 patients before or after renal transplantation residing in a single country. Each patient contributes only one serum sample in the analysis. A population view of linear correlations of hierarchically ordered fluorescence intensities reveals patterns in human immune responses with striking similarities with the previously described CREGs but also brings new information on the antigenic properties of class II HLA molecules. The same analysis affirms that “public” anti-DP antigenic responses are not correlated to anti DR and anti DQ responses which tend to cluster together. Principal Component Analysis (PCA) projections also demonstrate ordering patterns clearly differentiating anti DP responses from anti DR and DQ on several orthogonal planes. We conclude that a computer vision of human alloresponse by use of several dimensionality reduction algorithms rediscovers proven patterns of immune reactivity without any a priori assumption and might prove helpful for a more accurate definition of public immunogenic antigenic structures of HLA molecules. Furthermore, the use of Eigen decomposition on the Immune Response generates new hypotheses that may guide the design of more effective patient monitoring tests.

## Introduction

Multidimensional Descriptive Statistics are starting to play an increasingly important role in medical and wider scientific fields. Indeed, the integration of a classical clinical or biological phenotype with continuously increasing genetic information, or other complex biological markers such as microbiome analyses is challenging. Proportionately, the complexity of the data produced in current day-to-day clinical and scientific practice is growing. This further substantiates the necessity for data analysis algorithms that can either improve medical decisions or unveil unknown data properties important for clinical and scientific research (1, 2).

One of the most widely monitored human Immune Response is humoral anti HLA activity, which is usually measured in patients awaiting for a compatible transplant or patients that underwent transplantation and are at risk of developing antibodies against the foreign HLA of the graft. Measurement of anti HLA humoral response is frequently performed on Luminex platforms that can simultaneously define antibodies against a selection of ~100 different antigens coupled on fluorescent beads. These complex measurements are used as input to the organ allocation algorithms but are also used to determine Donor Specific Antibodies (DSA) that can endanger the long-term survival of the organ (3, 4).

A systematic view of cross reactivity for anti HLA Immune response goes back to 1964 when Van Rood reported the “group 4” antigens, to classify anti HLA B responses (5). Indeed, the very first observation is that frequently anti HLA B Immune Responses are directed against two mutually exclusive antigenic groups now known as Bw4 and Bw6 epitopes. Later on, Rodey and coworkers introduced the term of Cross-Reactive Groups (CREGs) of antigens based on frequency and intensity of specific patterns of Immune Responses (6). CREGs for HLA-A and B locus are well-established but CREGs for class II molecules are less definitive (7, 8). The main procedure for CREG definitions was based on a systematic view of complement dependent lymphocytotoxicity assays. A current view of CREGs is that they represent HLA proteins sharing at least one common antigenic epitope between the members of a CREG. CREG definitions and their counterparts called “serologic” specificities still have a substantial role in HLA Nomenclature.

Following the idea for a more precise description of HLA antigenic structures that are recognized from antibodies, El-Awar et al. used the position of single amino acid to define epitopic structures (9), while Duquesnoy and Marrari used a three linear amino acid sequence (triplets) in order to define functional HLA epitopes. Later, antigenic structures called eplet(s), were defined taking into account the three-dimensional structure of an HLA molecule (10). Nowadays, epitopic, eplet, and even the different electrostatic loads of amino acids between donors and recipients consist the basis of algorithms used for prediction of alloimmune responses in the transplantation field (11, 12).

Apart of the number of mismatched eplets that are highly associated with donor specific antibody formation, the number of mismatched donor HLA derived T cell epitopes that can be presented on recipients' HLA class II molecules can be calculated using the Predicted Indirectly ReCognizable HLA epitopes presented by recipient class II (PIRCHE-II) algorithm. PIRCHE-II scores are related to antibody formation and graft survival post transplantation (13).

However, formal unsupervised pattern recognition of antibody response in the Luminex era has not been fully undertaken yet. Indeed, Luminex platforms offer the possibility of simultaneous measurements of hundreds of antigenic reactions producing thus, providing a multidimensional view of a patient's immune response. We reasoned that being able to define clustering of the Ab response from numerous solid-state fixed antigens could be informative and may prove useful for better defining the public antigenic structures and/ or hidden cross reactivities.

Thus, we analyzed data coming from 1,748 serum samples from corresponding Immunized patients, before or post renal transplantation, with unsupervised machine learning algorithms. We employed two different data driven unsupervised descriptive statistic methods for extracting “hidden” information; usually neglected during the patient's workup. Both Spearman's Correlation Plots (14) and PCA (15) revealed several interesting patterns. These results are discussed in the perspective of basic properties of the Immune System in an alloreactivity setting but also on a basis for possible applications in transplantation practice.

## Materials and Methods

### Patients

All patients exhibiting anti HLA class II antibody activity and were attended during the last 5 years in the three major histocompatibility centers of Greece, have been included in the analysis. These were 1,748 patients, 56% males, 48 ± 13.6 years old consisting of 1,019 patients on the waiting list for renal transplantation with mean waiting time of 87 months and 729 renal transplant recipients of deceased (52%) or living donor (48%). The main immunosuppression regiment for renal transplant recipients consisted of induction therapy with anti-CD25 monoclonal antibody and maintenance therapy with mycophenolate acid (MPA)/calcineurin inhibitor (CNI)/corticosteroids. Positivity information was obtained either after using an LSM screening test from One Lambda (22,801 Roscoe Blvd West Hills USA) or after a historical background of positivity during the patient's follow up. The protocol was approved by the Health Research and Ethical Board of General Hospital of Athens “G.Gennimatas,” Hippokrateion Hospital of Thessaloniki and Evangelismos Hospital of Athens. Patients have given an informed consent for this study in accordance with the Declaration of Helsinki.

### Laboratory Tests

Positive sera were analyzed with Single Antigens anti-HLA Class II antibody detection kits from One Lambda on a Luminex 100 flow multicolor cytometer. To preserve as much homogeneity as possible only results obtained from LABScreen Single Antigen Class II kits (lots 10, 11, and 12) were included in the analysis, for having a consistent antigen-bead coupling. Laboratory tests were performed according to manufacturer's instructions and under the same experimental protocol across the 3 centers. All patients' sera were stored at −20°C until testing and EDTA treatment of sera was performed in all samples in order to prevent the prozone effect as previously published (16).

### Descriptive Statistics

In order to avoid redundancy in the analysis, we included just one serum sample per patient. In particular, when multiple sera were available for a patient, only the latest sample was taken into consideration for analysis. We could ultimately analyze immune responses from 1,019 patients on the waiting list for renal transplantation and 729 patients followed-up after the transplantation for the detection of Donor Specific Antibodies (DSA).

Correlation plots of Mean Fluorescence Intensities (MFI) measured for each antigen were created with Corrplot in R. For this analysis we used raw numerical values provided by the Luminex, hence avoiding the categorical variability (positive or negative) as is given by the manufacturer's analysis software.

PCA correlation plots were obtained with FactoMineR after scaling the raw data in the range of −1 to 1 (17). Thus, for each sample 96 unique reactions were measured corresponding to antibody responses against each antigen-coated bead.

## Results

**Figure 1:**
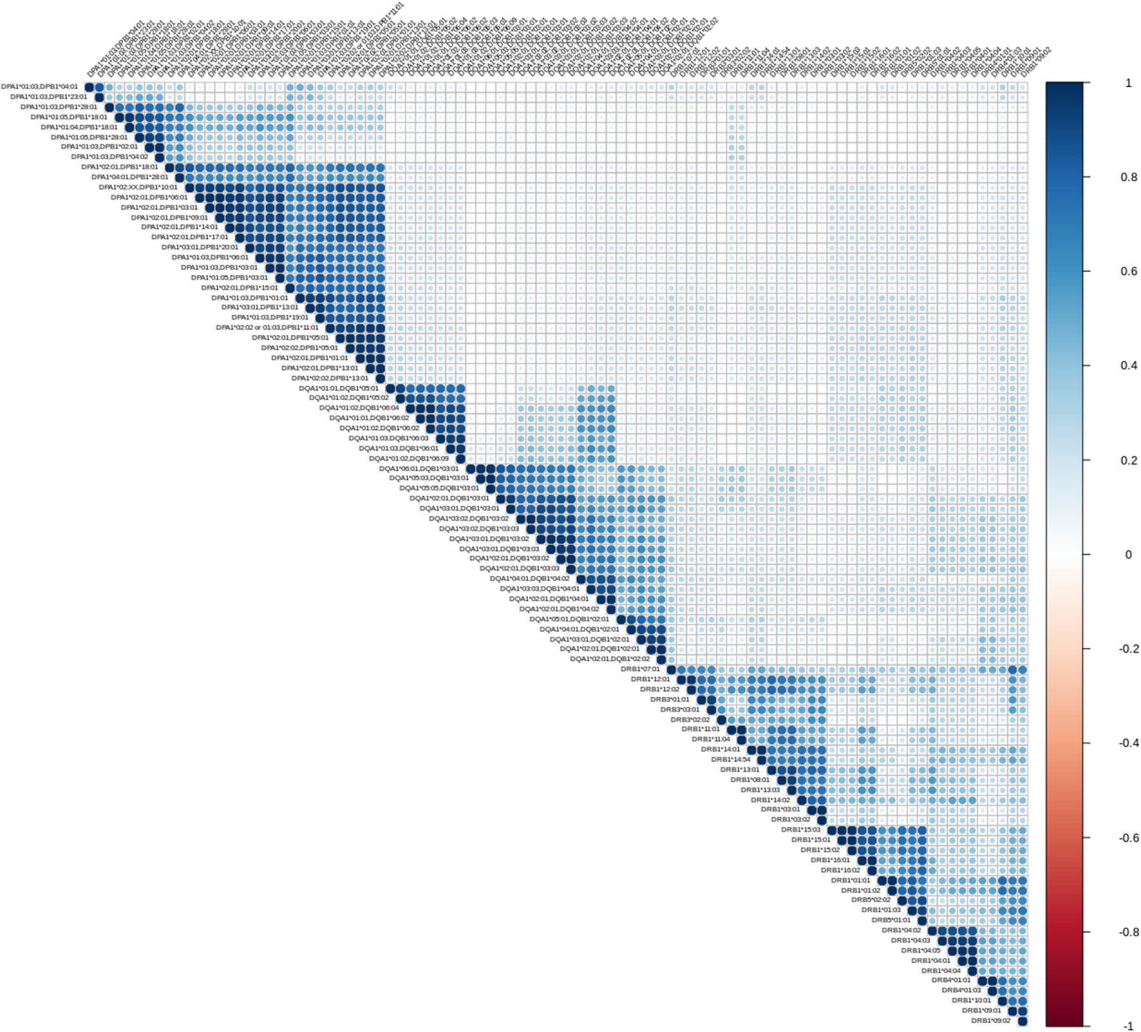
Correlation patterns of anti HLA class II immune responses from patients awaiting a transplant. Correlation coefficient of MFIs on 96 different antigen coated bead assays were computed among all beads and all sera. The left triangular panel represents pairwise correlations of unscaled MFIs among antigen-bead specific reactions. Similarly, the right panel is a color scale representing the coefficient correlation. *Rs* values (Spearman's correlation) range from −1 (dark red, negative correlation) to 0 (white, no correlation) and then up to 1 (dark blue, positive correlation). Agglomerative ordering was applied with a bottom to top ascending hierarchical clustering based on Mc Quitty distances of the dissimilarity matrix.

**Figure 2:**
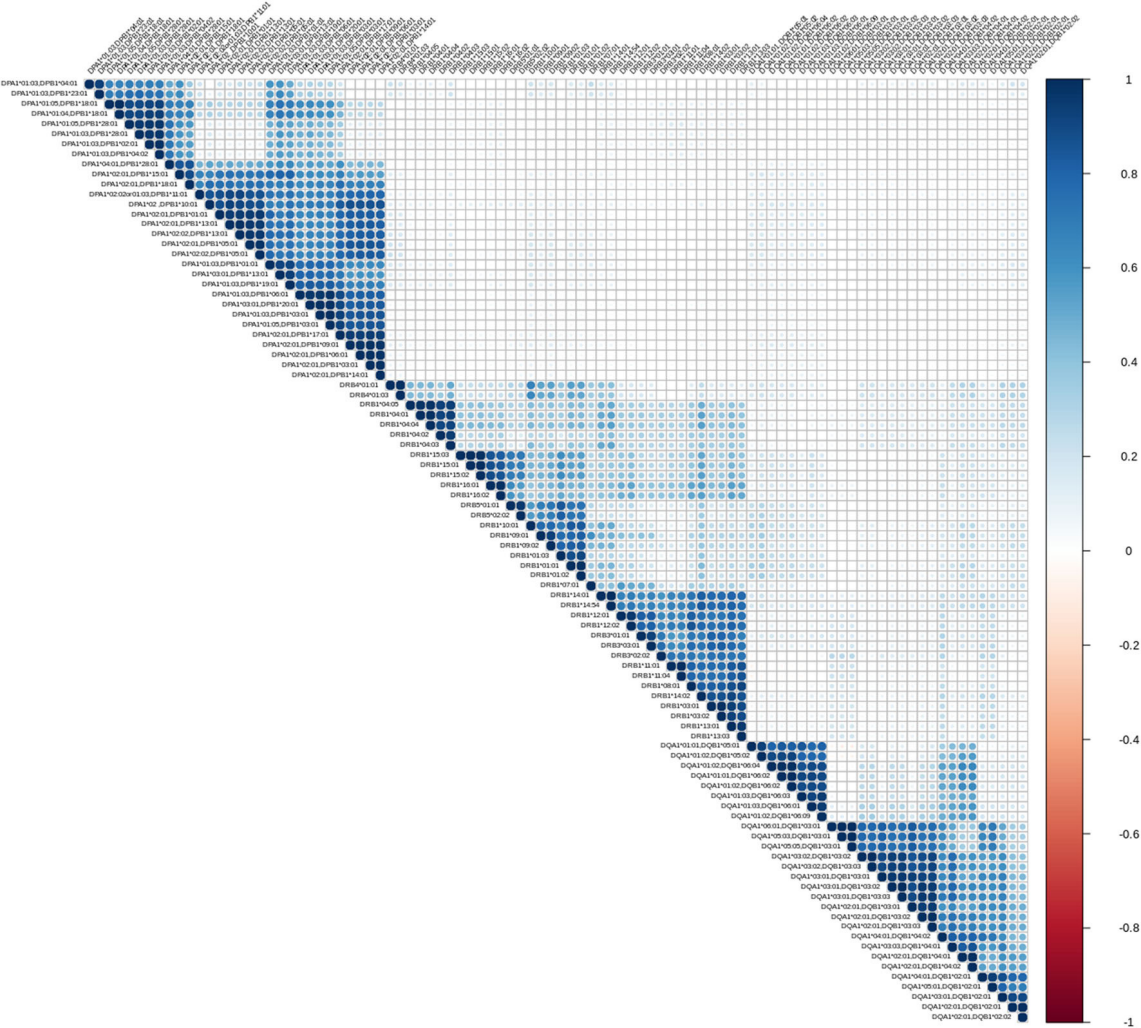
Correlation patterns of anti HLA class II immune responses from patients followed after transplantation. Correlation coefficient of MFIs on 96 different antigen coated bead assays were computed among all beads and all sera. The left triangular panel represents pairwise correlations of unscaled MFIs among antigen-bead specific reactions. Similarly, the right panel is a color scale representing the coefficient correlation. *Rs* values (Spearman's correlation) range from −1 (dark red, negative correlation) to 0 (white, no correlation) and then up to 1 (dark blue, positive correlation). Agglomerative ordering was applied with a bottom to top ascending hierarchical clustering based on Mc Quitty distances of the dissimilarity matrix.

The first exploratory analysis performed was the description of correlation coefficients between all reactions measured on a Luminex assay. Figure 1 shows a hierarchically ordered correlogram of MFIs for each antibody in a cohort of patients at the waiting list for transplantation while Figure 2 shows the analogous in patients after transplantation and under immunossupressive treatment.

Let us briefly explain the rationale of this first analysis. When two variables are considered to be correlated, a linear association between their corresponding observations is implied. The differentiation among positive and negative correlation depends on the type of their association. If positive correlation exists, when one variable increases, the other increases as well. Accordingly, for negatively correlated variables, when one increases, the other decreases. Finally, in case of zero correlation, it is implied that no relation between the two variables exists. For further elaboration, the fact that groups of correlated measurements are observed, implies the existence of clusters in human immune responses. One could therefore state that, these immune responses could be represented by their cluster representative.

From an immunological point of view the correlograms show several interesting patterns. Blocks with dark blue color (and most evident within each locus) reflect strong correlations between anti HLA class II responses. Blocks with light blue color reflect partial correlation of the responses. The white background blocks reflect uncorrelated immune responses. Not surprisingly there were no red blocks, suggesting the absence of overall negative correlations in anti HLA class II immune responses. It is therefore reasonable to assume that the clustered antibodies recognize HLA that share a public epitope which can be easily confirmed by looking at the amino acid sequences of these HLA antigens. According to that, a clear distinction between the immune responses against either DRB1,3,4,5, DQA1/DQB1, or DPA1/DPB1 alleles is apparent. Furthermore, within each locus we can easily see strong sub-clusterings analogous to previously described in CREGS or against the public functional epitopes referred as eplets. An example of inter locus crossreactivity is the strong correlation of responses between the alleles DQB1^*^ 05:01, 05:02, 06:01, 06:02, 06:03, 06:04, 06:09 (strong clustering) and DQB1 ^*^04:01, 04:02 as seen in Figures 1, 2. This crossreactivity can be explained from a shared epitope on beta chain at the position 52 proline (P) and 55 arginine (R). Also, responses against DRB1^*^11 show crossreactivity with 55 DE bearing DPB1 alleles (DPB1^*^02:01, ^*^04:02, ^*^18:01, ^*^28:01) providing an example of intra locus crossreactivity. These patterns confirm previous experimental studies but more importantly they may prove useful for addressing the question of which empirically defined epitopes are correct (18, 19).

As shown in Figures 1, 2, a remarkably similar correlation pattern is seen in sera coming from patients on the waiting list (Figure 1) as compared to those post-transplantation (Figure 2). Although, distance ordering of locus specific responses is different between the two groups i.e., proximity of DR vs. DP and DQ vs. DP, still the inter and intra locus immune response clustering remained unchanged.

Differential agglomerating ordering of the correlograms suggested that there is a difference in terms of relative proximity between DP vs. DR and DQ responses, among patients awaiting for transplantation and patients followed-up after transplantation. Indeed, DP responses are more distantly correlated with anti DR and DQ responses and agglomerate with either DR or DQ depending on the condition that the patient is either on a waiting list or evaluated after transplantation.

As it has been described in the previous section, applying some basic statistical analysis on the immune responses can lead to interesting remarks regarding the hidden relations between the observed variables. Although correlograms produce a comprehensive view of CREGs seen in the Greek Population, we wanted to test additionally whether other well-established methods for dimensionality reduction produce similar or complementary views of the Immune Response. We therefore used PCA, as this method uses the Eigen decomposition of the covariance matrix to represent both variable (antigen specificity) and individual (patient) responses, on successive orthogonal planes that maximize the variance of the observed object.

Moreover, the fact that we measure 96 immune response indices per patient, lead us to the question, whether we actually need all this information or there is a redundancy. A typical algorithm used in statistics to solve that problem is the PCA technique. PCA represents the data as points on a multidimensional space. Reducing the number of dimensions (planes) by presenting those that preserve most of the information is a common simplification practice.

In brief, PCA operates on the covariance matrix of the variables (similar to the correlation matrix) and computes the eigenvalues and eigenvectors of that matrix. Then, one can decide to keep only the eigenvectors corresponding to the largest eigenvalues for a meaningful interpretation. These eigenvectors represent the final space with the reduced dimensions upon which we will project our data in order to reduce the redundancy. The amount of information preserved (or variance explained in the new smaller space) can be inferred as the sum of the Eigen values related to the picked eigenvectors over the total sum of the eigenvalues.

**Figure 3:**
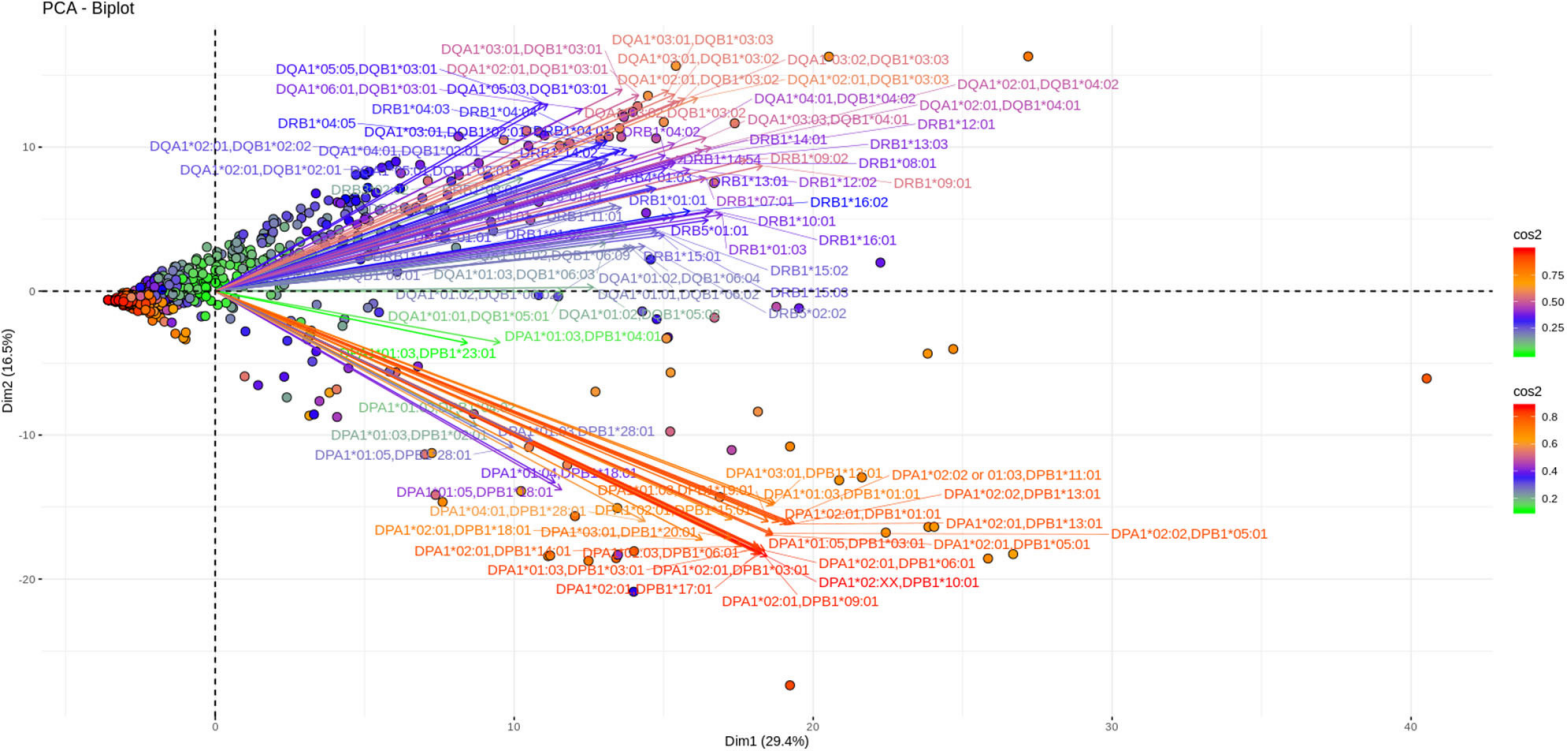
PCA biplots of anti HLA class II immune responses on patients immunized before the transplantation. Projections on Dim1 and Dim2. The points represent projections of individual reactions and arrows the corresponding variables according to the first and second principal components (referred to as Dim1 and Dim2 accordingly) of PCA. The color stripe on the right side exhibits the corresponding color vectors of explained variance, ranging from red color (indicating strong contribution on variance) to green (indicating weak contribution on variance).

**Figure 4:**
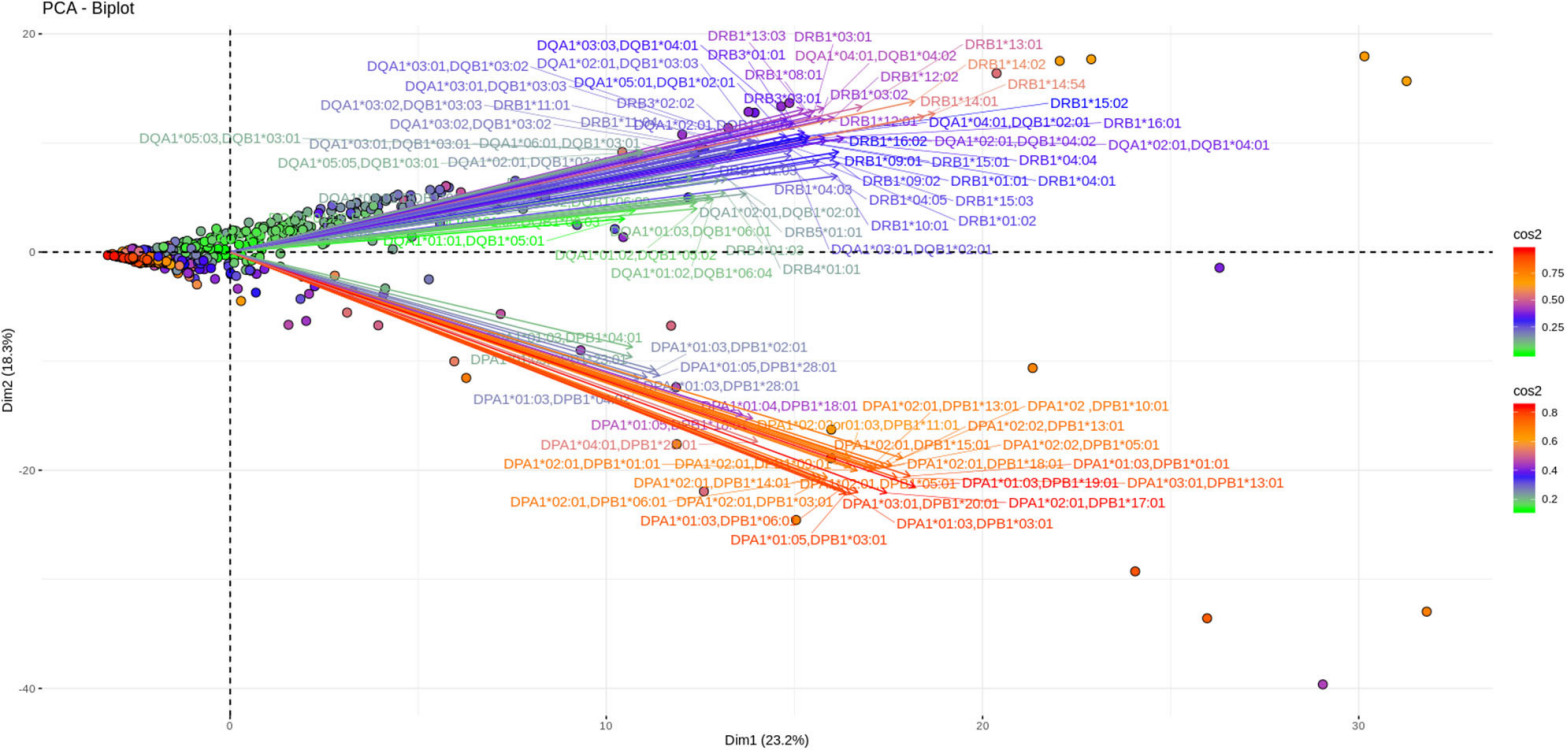
PCA biplots of anti HLA class II immune responses on patients after transplantation. Projections on Dim1 and Dim2. The points represent projections of individual reactions and arrows the corresponding variables according to the first and second principal components (referred to as Dim1 and Dim2 accordingly) of PCA. The color stripe on the right side exhibits the corresponding color vectors of explained variance, ranging from red color (indicating strong contribution on variance) to green (indicating weak contribution on variance).

As shown in Figures 3, 4, depicting dimension (Dim) 1 and 2 delimited plane, ~40% of the total variance is explained by the vectors corresponding to the two major “beams of arrows” of antigen specific response. The vectors shown in this graphic are the outcome of projecting the initially measured antigen specific responses to the 2D space as defined by the eigenvectors selected with the method described above. The angle between two arrows is proportional to coefficient correlations between them, while their lengths are proportional to the quantity of the explained variance. An anti DR and anti DQ “beam of arrows” is projected on the upper right quadrant of the PCA biplot while the anti DP “arrow beam” occupies the lower quadrant. The fact that the anti DP arrows are almost orthogonal to the corresponding anti DR and anti DQ arrows, suggest that in the Greek population the first variance explaining characteristic of anti HLA class II immune response is that anti DP responses are: (a) highly variable (long red vectors), (b) orthogonal to anti DR and DQ responses and, (c) important in terms of variance contribution. Taken together, these data suggest an absence of correlation among anti DP responses, on one hand, and anti DR and anti DQ on the other.

Furthermore, as each point on the plane is the projection of each serum measurement and the first plane explains most of the variance (Dim1 and Dim2 defined plane of the PCA), sera with a strong contribution to the overall variance (red colored points) are located apart from the origin of the axes while sera with small contribution to the variance (green color) are closer to the origin. Red points with projections far from the origin are well-explained on the Dim 1 and Dim 2 variance representing plane. However, some red points (the left part of the PCA biplot) are still close to the origin of axes suggesting that additional planes are necessary to capture their complete variance.

**Figure 5:**
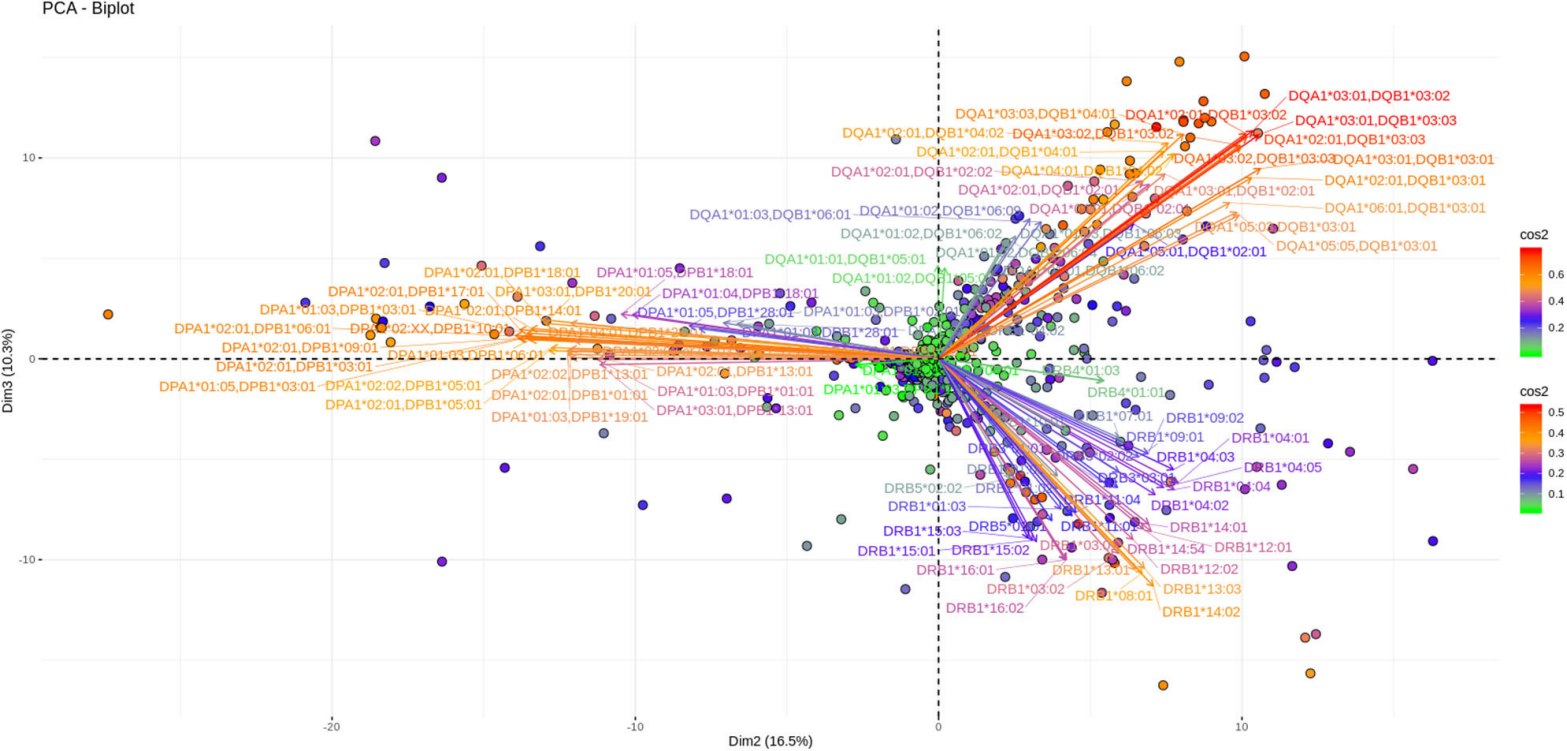
PCA biplots of anti HLA class II immune responses on patients immunized before the transplantation. Projections on Dim2 and Dim3. The points represent projections of individual reactions and arrows the corresponding variables according to the second and third principal components (referred to as Dim2 and Dim3 accordingly) of PCA. The color stripe on the right side exhibits the corresponding color vectors of explained variance, ranging from red color (indicating strong contribution on variance) to green (indicating weak contribution on variance).

**Figure 6:**
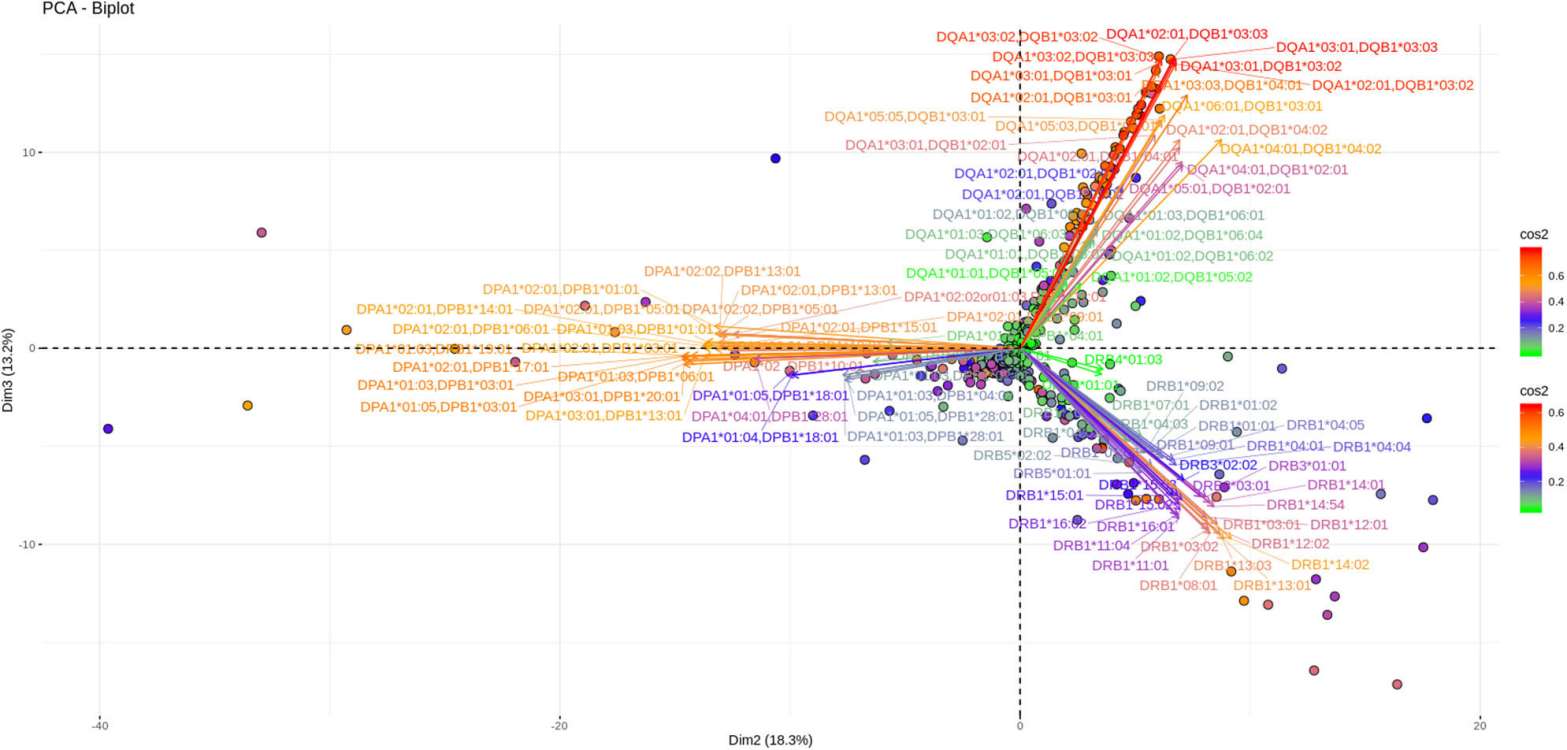
PCA biplots of anti HLA class II immune responses on patients after transplantation. Projections on Dim2 and Dim3. The points represent projections of individual reactions and arrows the corresponding variables according to the second and third principal components (referred to as Dim2 and Dim3 accordingly) of PCA. The color stripe on the right side exhibits the corresponding color vectors of explained variance, ranging from red color (indicating strong contribution on variance) to green (indicating weak contribution on variance).

Another 12% of the overall variance is depicted on Figures 5, 6 (Dim 2 and Dim 3 delimited plane). This plane represents the second most characteristic explaining variance pattern observed in the cohort. On this PCA biplot anti DP, anti DQ, and anti DR specific vectors form three “beams of arrows” with an angle of roughly 120° between them. Again, most of the points representing individual measurements and especially those with a high contribution to the variance are almost co-linear with the “beams of arrows” corresponding to the variables of each locus. This finding suggests that, compared to a frequent DR-DQ co-recognition, clear cut locus specific responses, have a relatively minor contribution to the total variance.

Noticeably equivalent patterns for both the vectors and the individual projections are observed in patients on the waiting list as well as in patients who receive immunosuppression after transplantation.

## Discussion

Alloimmune response against HLA class II molecules is a frequent laboratory measurement either before or after transplantation and is associated with graft injury and impaired graft function (20, 21). Although the HLA polymorphism and the complexity of the immune response determine the type of antibody production, shared-public functional antigenic determinants and close genetic linkage between antigens of different loci lead to the production of antibodies against specific antigenic groups. The definition of the reactivity patterns of these antibodies is useful in order to understand the principles of the alloresponse, to define the HLA immunogenic polymorphic residues, to design more effective laboratory tests and to improve allocation.

Agnostic methods play an important role in today's science. Epitope definition of HLA molecules with very few à priori assumptions was not attempted until very recently (22, 23). The field of hypothesis-free learning of the immune response starts growing. Simmons et al. (24) shows the advantages of searching patterns of reactivity in anti HLA class II responses. In this context we analyzed anti HLA Class II responses measured on the Luminex platform in a cohort of 1,748 patients monitored by the three major histocompatibility laboratories in Greece in order to identify reactivity patterns of these antibodies. In order to get the procedure as agnostic as possible we did not use cut off limits thus avoiding rendering each individual response strictly quantitative.

The observations in this study are based on “automatic” data driven classification of the alloimmune response performed with solid state fixed antigens. In order to generate the correlograms presented herein we tried several hierarchical ordering formulae. Only the application of general and/or Mc Quitty distance-based agglomerating models produced distinguishable locus specific responses, not only confirming known inter and intra locus cross reactivity among HLA antigens but also illustrating new features and providing important information regarding known or less studied loci.

All this information, coming from patients of a single country and tested under the same methodology, may become a hypothesis generating tool that can be used for formal modeling of the immune response. The example of anti HLA-DPB responses showing numerous undocumented but experimentally proven correlations both by Correlation and PCA analyses is an example of the utility of such tools.

Similar antibody reactivity patterns were observed between the patients awaiting an organ and those post transplantation. This observation indicates that beneath the complexity, the immune system reacts under a set of rules while not being affected by the immunosuppressive treatment.

On the contrary, a marking difference was observed at the uni-dimensional correlation pattern of anti HLA class II immune response when one focuses on pre or post transplantation patients. Indeed, the agglomerative hierarchical clustering of the correlograms differs in terms of inter locus proximity as DR and DQ responses are always ordered one next to the other while DP responses are considered as more proximal to DR or to DQ depending on the clinical condition. These results parallel our previous finding regarding a hierarchy in specific locus antigen recognition of the graft. Indeed, anti DQ graft specific antibodies have been described as the earliest *de novo* DSA usually seen during the follow up in renal transplant recipients in Greece (25, 26).

In order to get more insight on this “computer vision” of anti HLA class II response we further performed a PCA at the same population data. This unsupervised learning method transforms original data and visualizes them in subsequent orthogonal plains explaining the maximum of variance in terms of antibody specificity (variables) and responses in each serum (patients).

In this immune response setting, a three-dimension PCA explains about 55% of the population's total variance which is considered to be relatively limited in comparison with the classical thumb rule demanding an 80% variance explanation for a “good” PCA. However, this very preliminary analysis points to several hypotheses to be tested in the upcoming years. According to the PCA generated view, the first observation that comes from the Dim 1 and Dim2 variance plane, depicted in both patients' groups, is a differential clustering of DPB responses as compared to DRB and DQB. These responses appear to co-exist, confirming the analogous observation with the previously described correlograms. As a consequence, the overall picture of both types of analyses point to the same message in terms of no correlation between DPB and DR/DQ responses in the Greek population. Interestingly a similar finding was reported in a North American population (24). Therefore, a more careful analysis of DP specific responses could provide new markers for a safer organ allocation given that in Greece, but also in most European countries, anti DP responses are not taken into account for patient inscription on waiting lists. The second best explaining variance plane shows that another 12% of the variance is a clear mono locus immune response seen in both groups of patients. This can be interpreted as the second main characteristic of anti HLA class II immune response.

An important finding uncovered by this Machine Learning approach concerns the amount of immunologically important information that can be gained by the bead bound antigens proposed by the industry. Although the choice of these alloantigens is based on the relative frequencies of the alleles found in human populations, both the correlogram and the PCA results suggest that there is a lot of information redundancy in terms of antigen coupled beads. For instance, DRB1^*^15:01 and DRB1^*^15:02 specific reactions show a very strong correlation pattern and a quasi-identical PCA explained correlation and variance in several planes. This finding suggests that the information gained from the various DR15 coated beads appears equivalent. Therefore, if one measures the intensity of one DR15 coated bead, is then able to positively predict the values of the rest of the beads with a simple linear regression. Noticeably, an evidently similar pattern is also seen in reactions against DRB1^*^16:01 and 16:02, but also DRB1^*^14:01 and DRB1^*^14:54 coated beads. Probably, a formal regression analysis driven by PCA and/or correlogram clustering patterns could prove useful to indicate beads with redundant information. Vice versa antigens not incorporated into the Luminex display can be predicted by a cross reactive bead providing information for more informative HLA panel to monitor the allogeneic Immune response.

The information that emerges from this approach might prove useful for updating successful but still evolving matching algorithms. Several studies have shown that HLA alloantigen immunogenicity can be more accurately assessed by evaluating differences in the number and location of amino acid mismatches at continuous and discontinuous (eplet) positions, as well as their physicochemical properties. Actually, the HLA Matchmaker is widely used for prediction of harmful anti-graft immune responses and relies on the correct definition of eplets and eplet load differences between donor and recipient (27). More recently another approach based on differences in inter and intra locus donor-recipient HLA amino acid sequence along with an electrostatic mismatch score, enables prediction of allosensitization to HLA and also prediction of the risk of an individual donor-recipient HLA mismatch to induce DSA. However, the same study suggests that HLA Class II DSA responses are independently associated with these two HLA immunogenicity scores (12). An alternative approach for epitope based matching is the identification of the mismatched HLA derived epitopes that can be recognized by T cells via the indirect pathway using the PIRCHE-II algorithm. A higher PIRCHE-II score was related to both *de novo* donor specific HLA formation and allograft rejection (28). Our study proposes for an algorithm aiming to clarify the targets of the anti-HLA response after generation of effector alloantibodies. However, the generation of the antibodies requires triggering of a heterogeneous population of allopeptide specific CD4 T helper subsets that recognize different alloantigens and generate Ig-class switched alloantibody responses through the indirect CD4 T cells/allospecific B cells pathway. CD4 T cell alloresponse is a key determinant of transplant outcome provided that the indirect allorecognition pathway is activated in early acute rejection but also operates at late times points after transplantation sustaining chronic alloimmune responses. Therefore, the antibody specificity generated at different stages after transplantation is affected from the different epitopes that trigger different CD4 T helper subsets at varying stages of effector and memory differentiation. We consider that defining the targets of alloantibodies we may confirm the immunogenic T cell epitopes predicted in algorithms and study the dynamic of the phenomenon of intra and inter molecular epitope diversification or spreading (29).

Our approach may be used to enrich these algorithms and especially those based on eplet definitions. Indeed, empirically defined eplets can be experimentally validated or not by using the raw MFI values produced by the Luminex platforms and analyzed either through correlation or PCA based algorithms. However, all algorithms should always be considered in the context of other factors. We acknowledge that the results generated by the proposed algorithm are affected by immunological factors besides the HLA structures and the DSA such as hemodialysis—related immunosuppressive factors, heterologous immunity, immunological memory, immunosuppressive treatment and the development of regulatory mechanisms under immunosuppressive regiments. Under all these parameters the alloantibody profile of the patients is generated. This profile is essential for graft allocation and signals activation of anti-graft alloresponse post transplantation.

Having under consideration that a more accurate definition of the immunogenic HLA epitopes in different population groups will become a valuable tool for epitope matching, an important parameter for donor selection, here we propose a new approach to study the alloimmune response. From a transplantation perspective the use of a priori defined epitopes is currently widely discussed as a tool for allocation of organs as reviewed by Kramer et al. (30). We propose that a descriptive statistics approach can be another enlightening way to address antigenic proximities of HLA and helpful for the definition of frequent public epitopes present in a population. Furthermore, it has an enormous advantage since one can use data usually stored by transplantation laboratories and easily analyzable. It remains to be proven whether this type of analysis can delve deeper in the alloimmune study and define minimal regions of Immunogenicity based on experimental data to be used for organ allocation.

## Data Availability Statement

The raw data supporting the conclusions of this article will be made available by the authors, without undue reservation.

## Ethics Statement

The studies involving human participants were reviewed and approved by Health Research and Ethical Boards of Athens Gennimatas Hospital: 31563/19.10.17, Thessaloniki Hippokrateion Hospital: 291/21.02.2020, and Athens Evangelismos Hospital: 46/26-02-2020. The patients/participants provided their written informed consent to participate in this study.

## Author Contributions

AV, AF, ZT, KT, and GP performed the experiments, contributed to the design of statistical analyses, participated to the writing, and approved the final version. DG contributed to the conception of the statistical workflow, participated to the writing, and approved the final version. V-DK, MC, and CL contributed to the design of the study, participated to the writing, and approved the final version. ID, AI, and IT contributed to the design of the study and wrote the manuscript. All authors contributed to the article and approved the submitted version.

## Conflict of Interest

The authors declare that the research was conducted in the absence of any commercial or financial relationships that could be construed as a potential conflict of interest.

## Abbreviations

CREG: Cross-Reactive Group
Dim: Dimension
DSA: Donor Specific Antibodies
EDTA: Ethylenediaminetetraacetic Acid
HLA: Human Leukocyte Antigen
LSM: Labscreen mixed
MFI: Mean Fluorescence Intensity
PCA: Principal Component Analysis
PIRCHE-II: Predicted Indirectly ReCognizable HLA epitopes presented by recipient class II.
